# Determining the Optimal Cutoff Value for the Reverse Shock Index Multiplied by the Glasgow Coma Scale for the Prediction of In-Hospital Mortality in Pediatric Trauma Patients: A Retrospective Cohort Study

**DOI:** 10.3390/jcm14092994

**Published:** 2025-04-26

**Authors:** Sol Ji Choi, Min Joung Kim, Ha Yan Kim, Shin Young Park, Yoo Seok Park, Moon Kyu Kim, Ji Hwan Lee, Seo Hee Yoon

**Affiliations:** 1 Department of Pediatrics, Severance Children’s Hospital, Yonsei University College of Medicine, 50-1 Yonsei-ro, Seodaemun-gu, Seoul 03722, Republic of Korea; thfwl900803@yuhs.ac (S.J.C.); mkim@yuhs.ac (M.K.K.); 2 Department of Emergency Medicine, Yonsei University College of Medicine, 50-1 Yonsei-ro, Seodaemun-gu, Seoul 03722, Republic of Korea; boringzzz@yuhs.ac (M.J.K.); chilli89@yuhs.ac (S.Y.P.); pys0905@yuhs.ac (Y.S.P.); 3 Biostatistics Collaboration Unit, Department of Biomedical Systems Informatics, Yonsei University College of Medicine, 50-1 Yonsei-ro, Seodaemun-gu, Seoul 03722, Republic of Korea; hykim1213@yuhs.ac

**Keywords:** wounds and injuries, mortality, triage, pediatric emergency medicine

## Abstract

**Background/Objectives**: Despite the growing burden caused by pediatric trauma, the accuracy of prehospital triage remains suboptimal due to the lack of reliable pediatric-specific tools. In this study, we aimed to evaluate the predictive validity of the reverse shock index multiplied by the Glasgow Coma Scale (rSIG) for in-hospital mortality in pediatric trauma patients and to determine appropriate age-specific rSIG cutoff values for triage use. **Methods**: We conducted a multicenter retrospective observational study using data from the Korean Emergency Department-Based Injury In-Depth Surveillance registry; these data covered trauma patients aged ≤18 years, spanning the period from 2011 to 2022. The rSIG was calculated using the initial vital signs and Glasgow Coma Scale scores upon arrival at the emergency department. Age groups with shared rSIG cutoffs were identified using the area under the receiver operating characteristic curve (AUC) and Akaike information criterion. Cutoff values were derived using the Youden index or further optimized to align with triage goals (<5% under-triage, <35% over-triage). **Results**: Among 333,995 pediatric trauma patients, the in-hospital mortality rate was 0.07%. The rSIG cutoff values derived using the Youden index showed strong predictive performance, with an AUC of 0.920 (95% CI: 0.897–0.943). The cutoff values adjusted to meet triage goals—13.3 for those aged 0–9 years, 18.4 for 10–14 years, and 20.9 for 15–18 years—achieved the best balance, with 30.94% over-triage and 9.17% under-triage. **Conclusions**: The rSIG is a reliable predictor of in-hospital mortality in pediatric trauma cases. We recommend using cutoff values that are optimized to meet triage goals. Further research is warranted to develop standardized methods to derive triage-appropriate cutoff values.

## 1. Introduction

According to the World Health Organization, approximately 830,000 children die annually due to trauma, and it is estimated that half of those hospitalized for unintentional traumatic injuries develop lifelong disabilities [1]. Efforts to prevent traumatic pediatric injuries have been ongoing for decades [2]. However, a study by Mannix et al. in 2023 reported that the incidence of fatal injuries in children in the United States increased from 14.07 per 100,000 in 2011 to 17.30 per 100,000 in 2021, leading to a 250% increase in mortality due to pediatric trauma during the same period [3]. This highlights the urgent need for not only enhanced preventive strategies but also systematic improvements in pediatric trauma care systems in order to reduce trauma-related mortality.

The selection of an appropriate receiving hospital through prehospital triage is a critical step in trauma care, as the timely transport of severely injured pediatric patients to a specialized pediatric trauma center has been shown to improve clinical outcomes [4,5,6,7,8]. The American College of Surgeons (ACS) indicates that an ideal trauma triage tool should achieve an under-triage rate of <5% and an over-triage rate of <35% [9]. However, the performance of prehospital triage tools applicable to pediatric patients is suboptimal. A systematic review conducted by Lupton et al. (2022), which evaluated the field triage guidelines widely used in the United States, reported that the under-triage rate for children under 15 years of age ranged from 15.9% to 34.8%, far exceeding the recommended threshold [10]. This high under-triage rate may have contributed to delays in definitive care and an increased trauma-related mortality rate due to severely injured patients being directed to lower-level healthcare facilities.

One of the primary reasons for the low accuracy of field triage in pediatric trauma patients is the lack of reliable trauma triage tools that are applicable to this specific population. In a 2018 systematic review conducted by van der Sluijs et al., various field triage tools applicable to pediatric patients were evaluated; however, none met the performance standards outlined by the ACS [5]. According to this review, even the Pediatric Trauma Triage Checklist—the only field triage tool specifically designed for children—showed a high under-triage rate of 13.8%. These findings highlight the need for a field triage tool that enables the accurate identification of severely injured pediatric trauma patients.

In 2018, Kimura et al. proposed the use of the reverse shock index multiplied by the Glasgow Coma Scale (rSIG) to predict mortality in trauma patients [11]. The rSIG, which is calculated using the systolic blood pressure (SBP), heart rate (HR), and Glasgow Coma Scale (GCS), has since been evaluated in several follow-up studies and shown to be an effective predictor of massive transfusion requirements and trauma-related mortality in both adult and pediatric trauma populations [12,13,14]. However, for continuous variables such as the rSIG to be effectively used in triage settings, a reliable cutoff value is essential. To date, the literature contains varying suggested cutoffs for the identification of severe pediatric trauma, and no consensus has yet been established [7,15,16].

Therefore, this study was conducted with the aim of investigating the relationship between the rSIG and in-hospital mortality in pediatric trauma patients aged 18 years or younger and to determine appropriate rSIG cutoff values to predict in-hospital mortality. By determining a reliable cutoff with which to identify pediatric trauma patients who are at a high risk of death and applying it to field triage, it may be possible to reduce the number of preventable trauma-related deaths in children.

## 2. Materials and Methods

This was a multicenter, retrospective, observational study aimed at determining the optimal rSIG cutoff value to predict in-hospital mortality in pediatric trauma patients. The data were extracted from the Emergency Department-Based Injury In-Depth Surveillance (EDIIS) registry, a trauma surveillance system operated by the Korea Disease Control and Prevention Agency (KDCA), with 23 participating medical institutions. This study was approved by the KDCA and the Institutional Review Board of the Yonsei University Severance Hospital Ethics Committee (Approval No. 2024-3297-001). Artificial intelligence assistance in the form of OpenAI’s ChatGPT-4.0 was employed during the manuscript drafting process to improve clarity, grammar, and tone. No AI was used in the data analysis, statistical interpretation, or derivation of scientific conclusions.

### 2.1. Inclusion and Exclusion Criteria

This study included pediatric trauma patients aged 18 years or younger who received emergency care at EDIIS-affiliated institutions between 1 January 2011 and 31 December 2022. Acute trauma was defined as an injury event occurring within six hours before arrival at the emergency department [17,18]. The exclusion criteria stipulated the exclusion of patients with non-traumatic injuries (e.g., poisoning, drowning, burns), those missing core variables for the rSIG (e.g., SBP, HR, GCS), those with incomplete records regarding injury severity scores or treatment outcomes, those who underwent inter-hospital transfers before treatment completion, and those who were dead on arrival.

### 2.2. Study Data and Variables

The collected variables included demographic data (age, sex), the injury time, the arrival time, and the injury mechanism. The trauma severity variables included the excess mortality ratio-adjusted injury severity score (EMR-ISS), which is an injury severity metric based on the International Classification of Diseases 10 [19]. The initial vital signs and GCS were recorded upon arrival at the emergency department and were used to calculate the rSIG. Implausible values, such as SBP > 300 mmHg or <30 mmHg and HR > 200 bpm or <30 bpm, were treated as missing data. The primary outcome variable was in-hospital mortality.

### 2.3. Statistical Analysis

Categorical variables were reported as *n* (%), and group comparisons were performed using the chi-squared test. Continuous variables were reported as medians (interquartile range) due to their non-normal distributions and compared using the Mann–Whitney U test. A *p*-value < 0.05 was considered to indicate statistical significance. The statistical analysis was performed using R software, version 4.4.1 (http://www.R-project.org, accessed on 30 March 2025).

#### 2.3.1. Establishment of Age Groups with Shared rSIG Cutoffs

Among the variables comprising the rSIG, the normal reference ranges for SBP and HR vary according to age in pediatric patients. Consequently, the normal reference range for the rSIG was expected to differ by age, necessitating the determination of age groups with shared or similar rSIG cutoff values. The area under the receiver operating characteristic curve (AUC) and Akaike information criterion (AIC) values were calculated for all possible two-group and three-group classifications, and the results were visualized using a heatmap. The age group combination with the lowest AIC or highest AUC was selected to determine the optimal rSIG cutoff for each group.

#### 2.3.2. Determination of rSIG Cutoff Values

The rSIG cutoff values for each age group were determined using two approaches.

##### Youden Index (YI) Method

The YI (sensitivity + specificity − 1) is a widely used statistical tool for the determination of optimal cutoff values and has previously been applied in studies involving the calculation of rSIG cutoffs [13,20]. In this study, the YI was used to derive rSIG cutoff values for each age group. Receiver operating characteristic curves (ROCs) and AUC values were generated to compare the predictive performance of different rSIG cutoff values.

##### Optimization Based on over- and Under-Triage Rates

The ACS defines an optimal triage tool as having the following values: over-triage < 35% and under-triage < 5% [9]. However, the Cribari matrix, a conventional method used to assess the over- and under-triage rates, has been criticized for underestimating the under-triage rate in settings with a large proportion of minor injuries [21]. Given the low mortality rate (0.07%) in this study’s cohort, the method proposed by Peng et al. was adopted, defining the under-triage rate as the false-negative rate and the over-triage rate as the false-positive rate [21]. The rSIG cutoff was incrementally adjusted in 0.1 unit steps. The optimal cutoff was defined as the point where the sum of the under-triage and over-triage rates exceeding the recommended thresholds (5% and 35%, respectively) was minimal. If multiple cutoffs resulted in the same minimum sum, the final cutoff was chosen as the value with the lowest summed under-triage and over-triage rates.

## 3. Results

**Figure 1:**
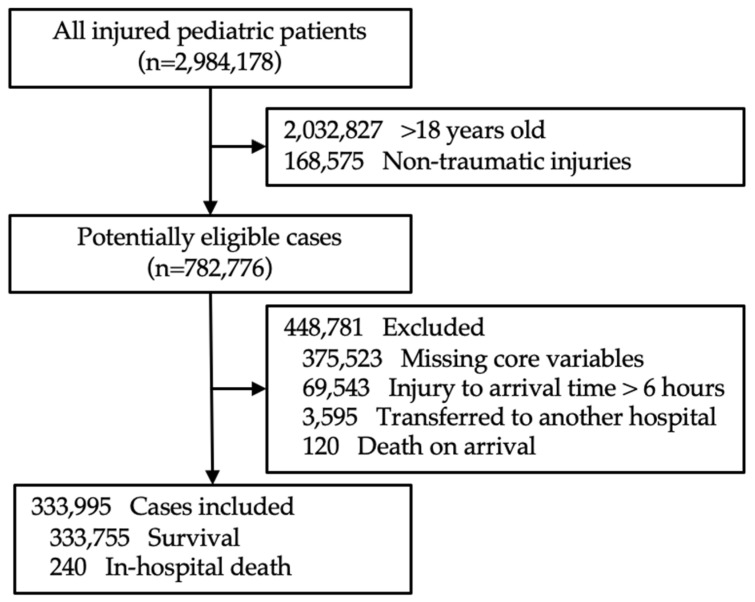
Flow diagram of the population selection process.

During the study period, a total of 2,984,178 patients visited 23 emergency medical centers due to various physical injuries. Among them, 2,032,827 patients were older than 18 years, and 168,575 were excluded due to non-traumatic injuries. Additionally, 375,523 cases had missing values for core variables, and 69,543 patients arrived at the hospital more than six hours after the injury. Consequently, data from 333,995 patients were included in the final analysis, with 240 in-hospital deaths, resulting in an in-hospital mortality rate of 0.07%. The case selection and exclusion processes are illustrated in Figure 1.

### 3.1. Characteristics of the Study Population

A comparison of the characteristics of the survival and in-hospital death groups is presented in Table 1. The in-hospital death group was older than the survival group and had a significantly greater proportion of traffic accident cases. Additionally, the period of time from injury to arrival was shorter in the in-hospital death group (*p* < 0.001 for all). The median rSIG was significantly lower in the in-hospital death group (4.67 [interquartile range 2.78, 8.62]) compared to the survival group (17.31 [interquartile range 14.10, 21.15]). Significant differences between the two groups were also observed for injury severity indicators such as the GCS and EMR-ISS (*p* < 0.001 for all).

### 3.2. Age Groups with Shared rSIG Cutoffs

**Figure 2:**
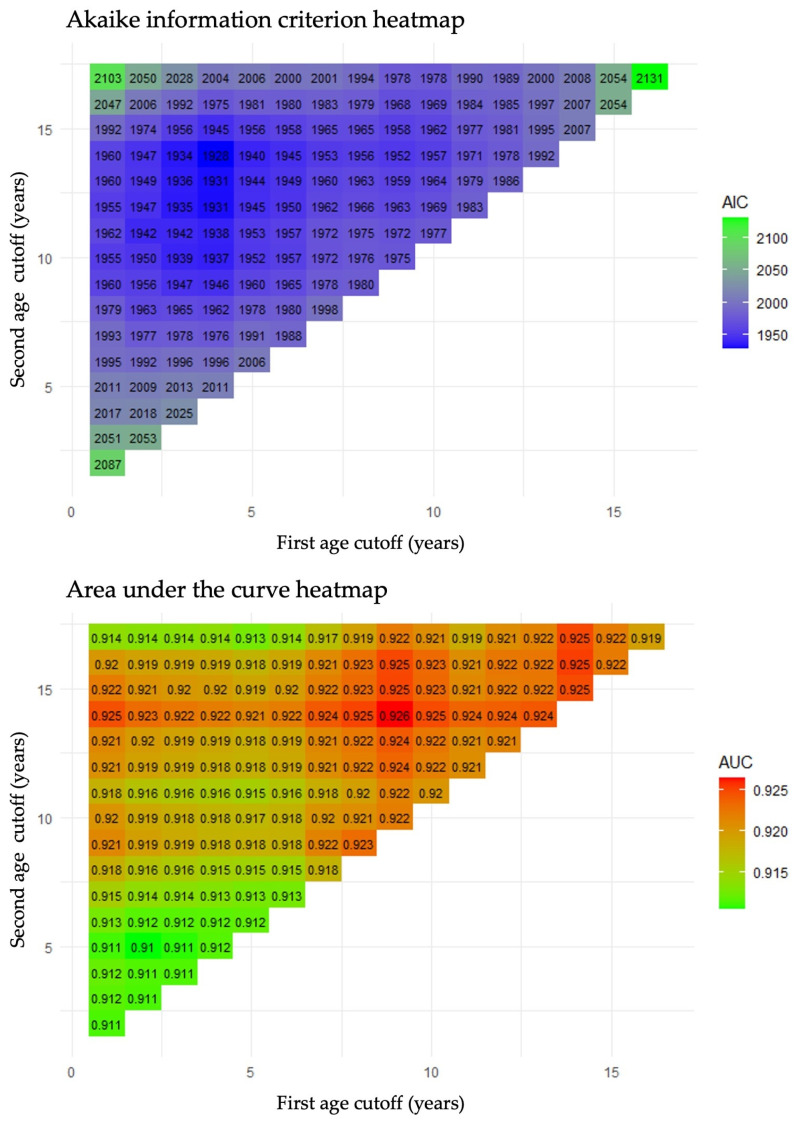
Heatmap of predictive performance regarding in-hospital mortality across age group combinations. In the AUC heatmap (**bottom**), colors closer to red indicate stronger discriminatory performance, whereas in the AIC heatmap (**top**), darker shades of blue indicate a better model fit.

Figure 2 illustrates how different combinations of two- and three-group age cutoffs influence the predictive performance of the rSIG for in-hospital mortality, presented in the form of AIC (top) and AUC (bottom) heatmaps. In the AUC heatmap, colors closer to red indicate a stronger discriminatory performance, whereas, in the AIC heatmap, darker shades of blue indicate a better model fit. When dividing the population into two age groups, the optimal age range to achieve the highest AUC (0.925) was ≤14 years, while the age range that achieved the lowest AIC (1977) was ≤10 years. When using three age groups, the optimal ranges for the highest AUC (0.926) were ≤9 years and ≥15 years, whereas the boundaries for the lowest AIC (1928) were ≤4 years and ≥15 years.

Based on these results, we adopted two group classifications: (1) two-group classifications (0–10 years and 11–18 years, or 0–14 years and 15–18 years) and (2) three-group classifications (0–4 years, 5–14 years, and 15–18 years, or 0–9 years, 10–14 years, and 15–18 years). Additionally, we derived rSIG cutoff values for the previously established age classifications (0–6 years, 7–12 years, and 13–18 years), which were developed based on the pediatric age-adjusted shock index (SIPA), and we compared the predictive performance regarding in-hospital mortality across different age classification methods [22].

### 3.3. Optimal rSIG Cutoff for Maximization of AUC Using YI

The rSIG cutoff values derived using the YI and the AUC for each cutoff are presented in Table 2. The rSIG cutoff values were higher in groups with older age ranges. The AUC for the rSIG cutoff in each age group was lowest in the group aged 0–10 years, with an rSIG cutoff of <9.643 and an AUC cutoff of 0.847 (95% confidence interval [CI]: 0.797–0.896), while the highest AUC was observed in the group aged 7–12 years, with an rSIG cutoff of <12.089 and an AUC of 0.949 (95% CI: 0.926–0.972). Overall, groups that included children under 5 years of age tended to have lower AUC values. However, even in these groups, all AUC values exceeded 0.8, demonstrating clinically significant predictive power. Furthermore, groups that primarily consisted of patients aged 5 years and older exhibited AUC values greater than 0.9, indicating excellent diagnostic performance.

**Figure 3:**
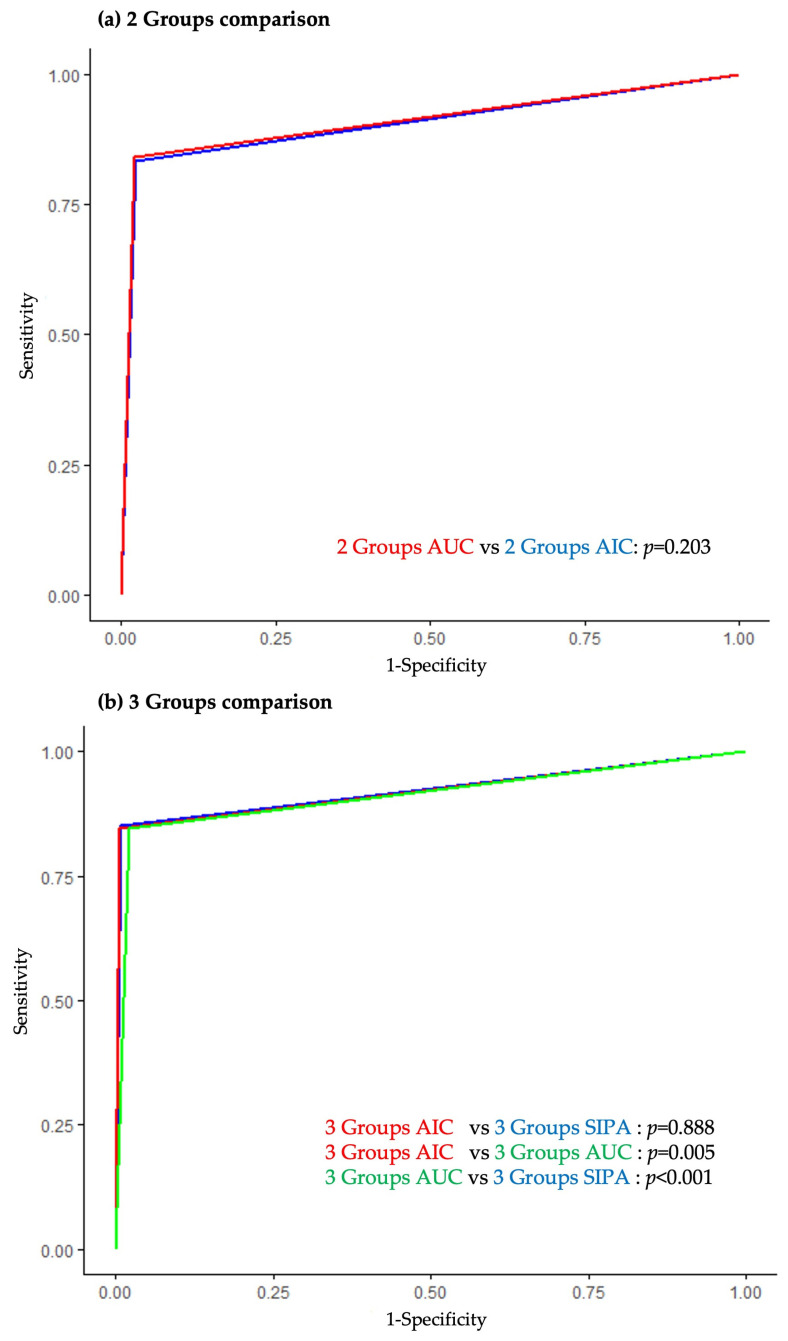
ROCs comparing the predictive performance of the rSIG for in-hospital mortality across different age group classifications. (**a**) In the two-group model, no significant difference was observed between cutoffs derived using the YI from age classifications based on the AUC and AIC. (**b**) In the three-group model, cutoffs derived from the AUC-based age classification showed significantly poorer performance compared to those from the AIC-based and SIPA-based classifications. AUC values for each model are presented in Table 2.

The ROC and AUC comparisons for each rSIG cutoff are presented in Figure 3. In the two-group model, the rSIG cutoff derived using the YI from the age group classification based on the AUC yielded an AUC of 0.910 (95% CI: 0.887–0.933), which was slightly higher than that derived from the AIC-based age group classification (AUC 0.905; 95% CI: 0.881–0.928), although the difference was not statistically significant (*p* = 0.203). In the three-group model, the rSIG cutoff from the AUC-based age group classification demonstrated a significantly lower AUC (0.912; 95% CI: 0.889–0.934) compared to both the AIC-based age group classification and the SIPA-based classification (both with an AUC of 0.920; 95% CI: 0.897–0.943).

### 3.4. Optimal rSIG Cutoff Based on Over-Triage and Under-Triage Rates

Table 3 presents the rSIG cutoff values for each age group, optimized according to the under-triage/over-triage rates in accordance with the triage tool targets set by the ACS, along with the corresponding under-triage and over-triage rates.

For age groups consisting of patients aged 5 years and older, it was possible to determine cutoff values that were closely aligned with the triage tool’s targets of an under-triage rate below 5% and an over-triage rate below 35%. However, for groups that included patients under 5 years of age, an appropriate balance between under-triage and over-triage could not be identified. In these age groups, the under-triage rate exceeded the target by three- to five-fold, indicating substantial difficulty in establishing an appropriate balance. After applying the respective cutoffs to the designated age groups and evaluating the combined performance across the entire age range, the rSIG’s overall performance was assessed. The results demonstrated that the over-triage rate generally met the target value, while the under-triage rate exceeded the target, ranging from 9.17% to 10.83%. Among the cutoff values derived from various age group combinations, the cutoffs obtained through the cohort’s division into three age groups based on the AUC demonstrated the lowest combined exceedance of the acceptable thresholds for under-triage and over-triage.

## 4. Discussion

This study confirms that rSIG is an effective tool for the prediction of in-hospital mortality in pediatric trauma patients. The age-specific cutoffs for the rSIG, derived using the YI, were 7.558 for ages 0–4 years, 10.142 for ages 5–14 years, and 13.507 for ages 15–18 years. Alternatively, they were 7.558 for ages 0–6 years, 12.089 for ages 7–12 years, and 14.507 for ages 13–18 years. When using these cutoffs, the model demonstrated excellent predictive performance, with an AUC of 0.920 (95% CI: 0.897–0.943). In contrast, when the cutoffs were optimized based on the over-/under-triage rates, they were 13.3 for ages 0–9 years, 18.4 for 10–14 years, and 20.9 for 15–18 years, achieving a balanced performance, with an over-triage rate of 30.94% and an under-triage rate of 9.17%. Although this under-triage rate does not meet the ACS requirement of less than 5%, it is superior to those of previously reported pediatric-specific field triage tools, which demonstrated under-triage rates ranging from 12.7% to 50.9%, as reported by van der Sluijs et al. [5]. Therefore, we consider the combination of this age group classification and its corresponding cutoff values to be the most balanced and most closely aligned with the goals of field trauma triage among the various cutoff strategies proposed in this study.

To further support the clinical application of these findings, we present a hypothetical example illustrating how age-specific rSIG cutoff values may influence triage decisions in practice. Let us consider a pediatric patient who was struck by a car as a pedestrian and who presents with an rSIG of approximately 15 based on their initial vital signs and GCS. If the patient is 13 years old, this rSIG value would fall below the age-specific cutoff of 18.4, suggesting a high risk of in-hospital mortality. In contrast, if the same rSIG was observed in a 3-year-old child, it would exceed the age-specific cutoff of 13.3, indicating a lower risk. Accordingly, in this scenario, the 13-year-old patient would be prioritized for transport to a pediatric trauma center over the 3-year-old patient despite having the same rSIG value. This example illustrates how the application of age-adjusted rSIG cutoff values can help prevent potential misclassification and enable more appropriate resource allocation in pediatric trauma triage.

In this study, the rSIG cutoff values were derived based on vital signs measured upon arrival at the emergency department. However, the rSIG is also intended for use in prehospital triage. Therefore, concerns may arise regarding potential discrepancies between the prehospital and in-hospital vital signs and how these differences could affect the rSIG value. In a 2020 study by Trust et al., the agreement between the prehospital and emergency department vital signs was evaluated in trauma patients. The authors reported strong agreement for the GCS (intraclass correlation coefficient = 0.79) and a fair-to-moderate agreement for the HR (intraclass correlation coefficient = 0.59) and SBP (intraclass correlation coefficient = 0.48) [23]. Based on these findings, the authors concluded that “despite challenges in prehospital assessments, the field GCS, SBP, and HR correlate well with the first ED vital signs”, supporting the notion that the components used to calculate the rSIG may exhibit reasonable stability between these two settings. These findings indicate the potential for the rSIG cutoff values proposed in this study to be preliminarily applied in the prehospital setting. Nevertheless, further studies are needed to derive and validate rSIG cutoffs directly using prehospital data.

The previous rSIG studies conducted by Repucci et al. and Lammers et al. utilized age groupings derived from the SIPA and determined cutoffs using the YI [7,12,22]. While effective, this approach prompts two critical questions. Firstly, several studies have reported that pediatric patients with traumatic brain injuries may exhibit age-dependent differences in prognosis [24,25]. However, the age group classifications obtained using the SIPA do not account for variations in the GCS, which is a key component of the rSIG. This limitation suggests that SIPA-based groupings may be suboptimal when applying the rSIG in pediatric populations. Therefore, we considered the need for age groupings specifically optimized for the rSIG. Among the five age group classifications evaluated, when comparing the rSIG cutoffs derived using the YI across each classification, both the SIPA classification and an AIC-derived three-group model showed the strongest predictive performance (AUC 0.920), supporting the continued use of the SIPA classification for the rSIG.

Secondly, while the YI has been widely used to determine cutoff values for various trauma triage tools, triage goals are fundamentally focused on minimizing under-triage—even at the cost of moderate over-triage. This raises the question of whether the YI is appropriate to derive cutoffs that align with these triage goals. Table 4 presents the under-triage and over-triage rates associated with the rSIG cutoff values derived using the YI. These cutoffs were found to result in a lower over-triage rate but a relatively high under-triage rate, which contradicts the intended objective of trauma triage tools. Therefore, cutoff values based on the YI cannot be considered to be optimized for the specific goals of trauma triage. As such, we recommend using cutoff values based on the under- and over-triage rates, rather than those derived from the YI, as a more appropriate tool for field triage. While we do not consider our alternative method to be definitive, we highlight the need for further research to establish a consensus-based approach that better aligns with triage system goals.

In their study, Lammers et al. also aimed to establish rSIG cutoff values using mortality as the outcome variable, similar to the present study [12]. When comparing the cutoff values derived using the same statistical tool—the YI—the cutoffs obtained in our study were generally higher than those reported by Lammers et al. This discrepancy may be attributed to differences in the characteristics of the study populations. While our study included pediatric trauma patients from non-conflict regions who sustained injuries during daily life, Lammers et al. focused on pediatric patients who were injured in conflict zones—a group that is likely to have a greater proportion of severe trauma cases [12].

Age groups that included children under 5 years demonstrated relatively low predictive performance compared to those composed of children aged 5 years and older. In these younger groups, the under-triage rates significantly exceeded the recommended thresholds. Interestingly, the under- and over-triage rates calculated using statistical values in the study by Lammers et al. were 24% and 20%, respectively, indicating an elevated under-triage rate in younger children, which is consistent with our findings.

Two potential factors may account for this phenomenon. Firstly, in infants and young children, hypotension typically presents as a late sign in cases of hypovolemic shock [26]. As a result, the SBP may remain within the normal range during early shock, potentially leading to an inappropriately high rSIG score and these patients’ subsequent misclassification as low risk. Secondly, accurately assessing the GCS in preverbal or very young children can be challenging. In the work of Caruana et al., acceptable consistency in GCS scoring (Cronbach’s alpha = 0.78) was achieved only among clinicians and nurses with more than five years of experience, suggesting that a reliable GCS assessment is highly dependent on clinical experience [27]. However, such expertise is not always available in prehospital or general emergency settings. DiBrito et al. also reported that the motor component of the GCS demonstrated the highest inter-rater agreement compared to the verbal and eye components [28]. In light of these findings, the use of simplified alternatives may be worth exploring, such as the reverse shock index multiplied by the motor component of the GCS, which has recently been evaluated in pediatric trauma populations by Smida et al. [4]. Further studies are warranted to validate its applicability in this context.

While the GCS remains the cornerstone of neurological assessment in pediatric trauma, it is not sufficient to guide triage decisions when used alone, particularly in cases of suspected traumatic brain injury. According to the National Institute for Health and Care Excellence guidelines, community healthcare providers are advised to refer patients with signs such as neurological deficits, loss of consciousness, skull fractures, or penetrating head injuries directly to emergency hospital departments, using ambulance transport when necessary [29]. These recommendations highlight that effective triage for pediatric traumatic brain injury must incorporate broader clinical and mechanistic factors beyond the GCS or rSIG alone. In this context, while indices such as the rSIG may support early risk stratification, their use should be integrated with comprehensive clinical judgment tailored to the mechanism of injury and presenting features.

This study has several limitations. Firstly, the data were collected from a single country, which limits the generalizability of the findings to other regions. Secondly, a substantial number of eligible cases were excluded due to missing values in key variables, which may have introduced selection bias and affected the results. Thirdly, although this study was intended to inform prehospital triage, all rSIG values were calculated based on hospital-level measurements, which may differ from those obtained in the field. While the relevant literature suggests reasonable agreement for the components of the rSIG, further validation using prehospital data is warranted. Fourthly, as noted earlier, the method used in this study to determine cutoff values has not yet been standardized or widely agreed upon. Future research should focus on developing optimized and standardized methods to derive cutoffs that are tailored to the specific goals of trauma triage tools. Finally, the extremely low in-hospital mortality rate in our dataset (0.07%) represents a statistical limitation. Although the overall sample size was large, the small number of deaths may affect the stability and generalizability of the proposed rSIG cutoffs. Further validation using independent datasets with higher event rates is warranted to confirm their predictive utility.

## 5. Conclusions

This study demonstrates that the rSIG is a reliable predictor of in-hospital mortality in pediatric trauma patients. While the cutoff values derived using the YI showed strong predictive performance, those that were optimized based on triage goals provided a more balanced approach and aligned with clinical priorities. We recommend using optimized age-specific rSIG cutoffs to enable acceptable over- and under-triage rates and, thus, enhance the accuracy of field triage. Further research is required to obtain standardized cutoff derivation methods that are suitable for pediatric trauma systems.

## Figures and Tables

**Table 1:** Characteristics of the study population.

Variable	Survival Group (n = 333,755)	In-Hospital Death Group (n = 240)	*p*-Value
Age, years	7 (3, 13)	15 (6, 17)	<0.001
Male, n (%)	224,200 (67.2)	175 (72.9)	0.058
Injury mechanism, n (%)			<0.001
Traffic accident	47,275 (14.2)	145 (60.4)	
Fall	130,166 (39.0)	87 (36.3)	
Blunt injury	123,011 (36.9)	6 (2.5)	
Penetrating injury	33,064 (9.9)	2 (0.8)	
Other ^a^	239 (0.1)	0 (0.0)	
Time from injury to arrival (min)	60 (30, 94)	41 (26, 94)	<0.001
Arrived by ambulance, n (%)	46,142 (13.8)	165 (68.8)	<0.001
SBP (mmHg)	110 (100, 125)	110 (84, 130)	0.043
DBP (mmHg, n= 333,155)	68 (60, 78)	70 (55, 84)	0.346
HR (beats/min)	98 (85, 112)	110 (84, 134)	<0.001
RR (breaths/min, n = 333,142)	20 (20, 24)	20 (18, 26)	0.059
BT (°C, n = 333,583)	36.6 (36.4, 36.8)	36.2 (36.0, 36.6)	<0.001
GCS	15 (15, 15)	4 (3, 8)	<0.001
rSIG	17.31 (14.10, 21.15)	4.67 (2.78, 8.62)	<0.001
EMR-ISS	9.0 (4.0, 9.0)	50 (25, 66)	<0.001

Frequency (%) or median (interquartile range); SBP, systolic blood pressure; DBP, diastolic blood pressure; HR, heart rate; RR, respiratory rate; BT, body temperature; GCS, Glasgow Coma Scale; rSIG, reverse shock index multiplied by the Glasgow Coma Scale; EMR-ISS, excess mortality ratio-adjusted injury severity score. ^a^ Other included low-frequency injury mechanisms, such as injury by a machine.

**Table 2:** Optimal rSIG cutoff for maximization of AUC using YI.

	Group 1			Group 2			Group 3			Overall
Age Group	Age Range (years)	Cutoff	AUC (95% CI)	Age Range (years)	Cutoff	AUC (95% CI)	Age Range (years)	Cutoff	AUC (95% CI)	AUC (95% CI)
2 Groups AUC	0–14	9.643	0.872 (0.833–0.912)	15–18	13.507	0.946 (0.920–0.971)	-	-	-	0.910 (0.887–0.933)
2 Groups AIC	0–10	9.643	0.847 (0.797–0.896)	11–18	13.507	0.945 (0.923–0.968)	-	-	-	0.905 (0.881–0.928)
3 Groups AUC	0–9	9.643	0.850 (0.799–0.901)	10–14	12.092	0.931 (0.876–0.985)	15–18	13.507	0.946 (0.92–0.971)	0.912 (0.889–0.934)
3 Groups AIC	0–4	7.558	0.850 (0.781–0.918)	5–14	10.142	0.946 (0.92–0.971)	15–18	13.507	0.946 (0.92–0.971)	0.920 (0.897–0.943)
3 Groups SIPA	0–6	7.558	0.850 (0.793–0.908)	7–12	12.089	0.949 (0.926–0.972)	13–18	13.507	0.911 (0.846–0.977)	0.920 (0.897–0.943)

rSIG, reverse shock index multiplied by the Glasgow Coma Scale; AUC, area under the curve; YI, Youden index; 95% CI, 95% confidence interval; AIC, Akaike information criterion; SIPA, pediatric age-adjusted shock index.

**Table 3:** Optimal rSIG cutoff based on over-triage and under-triage rates.

	Group 1			Group 2			Group 3			Overall
Age Group	Age Range (years)	Cut Off	Over-Triage Rate Under-Triage Rate	Age Range (years)	Cut Off	Over-Triage Rate Under-Triage Rate	Age Range (years)	Cut Off	Over-Triage Rate Under-Triage Rate	Over-Triage Rate Under-Triage Rate
2 Groups AUC	0–14	14.8	35.87 15.18	15–18	20.9	35.64 5.47	-	-	-	35.82 10.00
2 Groups AIC	0–10	13.3	28.04 20.25	11–18	19.3	28.07 5.59	-	-	-	30.78 10.42
3 Groups AUC	0–9	13.3	29.57 18.92	10–14	18.4	30.62 2.63	15–18	20.9	35.64 5.47	30.94 9.17
3 Groups AIC	0–4	12.2	29.83 22.73	5–14	16.7	33.99 10.29	15–18	20.9	35.64 5.47	33.78 10.00
3 Groups SIPA	0–6	12.6	29.96 22.95	7–12	16.1	23.60 9.68	13–18	19.3	23.65 6.08	29.62 10.83

AUC, area under the curve; AIC, Akaike information criterion; SIPA, pediatric age-adjusted shock index.

**Table 4:** Over- and under-triage rates of the rSIG cutoff values derived using the Youden index.

	Group 1			Group 2			Group 3			Overall
Age Group	Age Range (years)	Cutoff	Over-Triage Rate Under-Triage Rate	Age Range (years)	Cutoff	Over-Triage Rate Under-Triage Rate	Age Range (years)	Cutoff	Over-Triage Rate Under-Triage Rate	Over-Triage Rate Under-Triage Rate
2 Groups AUC	0–14	9.643	2.3323.21	15–18	13.507	1.529.38	-	-	-	2.1815.83
2 Groups AIC	0–10	9.643	2.8327.85	11–18	13.507	1.619.32	-	-	-	2.4115.42
3 Groups AUC	0–9	9.643	3.0027.03	10–14	12.092	0.7013.16	15–18	13.507	1.529.38	2.2715.42
3 Groups AIC	0–4	7.558	0.5231.82	5–14	10.142	0.3317.65	15–18	13.507	1.529.38	0.6215.83
3 Groups SIPA	0–6	7.558	0.4131.15	7–12	12.089	1.5919.35	13–18	13.507	1.438.78	0.9915.83

AUC, area under the curve; AIC, Akaike information criterion; SIPA, pediatric age-adjusted shock index.

## Data Availability

The datasets presented in this article are not readily available because they are owned by the KDCA, which prohibits the disclosure of data collected through the EDIIS without prior permission. Requests to access the datasets should be directed to the KDCA (https://www.kdca.go.kr), which requires appropriate approval for academic use.

## Abbreviations

The following abbreviations are used in this manuscript:

ACS	American College of Surgeons
rSIG	Reverse shock index multiplied by the Glasgow Coma Scale
SBP	Systolic blood pressure
HR	Heart rate
GCS	Glasgow Coma Scale
EDIIS	Emergency Department-Based Injury In-Depth Surveillance
KDCA	Korea Disease Control and Prevention Agency
EMR-ISS	Excess mortality ratio-adjusted injury severity score
AUC	Area under the receiver operating characteristic curve
AIC	Akaike information criterion
ROC	Receiver operating characteristic curve
YI	Youden index
DBP	Diastolic blood pressure
RR	Respiratory rate
BT	Body temperature
95% CI	95% confidence interval
SIPA	Pediatric age-adjusted shock index
